# Epidemiology of foot complaints in systemic lupus erythematosus

**DOI:** 10.1186/1757-1146-8-S2-O30

**Published:** 2015-09-22

**Authors:** S Otter, S Kumar, P Gow, N Dalbeth, KA Davies, S Panthakalam, K Rome

**Affiliations:** 1 Auckland University of Technology, Auckland, New Zealand; 2 Middlemore Hospital, Papatoetoe 2025, New Zealand; 3 The University of Auckland, Auckland, 1010, New Zealand; 4 Brighton & Sussex Medical School, Brighton, BN1 9PX, UK; 5 Eastbourne District General Hospital, Eastbourne, BN21 2UD, UK

**Keywords:** Systemic Lupus Erythematosus, Foot, Epidemiology

## Background

Foot complaints are common in inflammatory arthropathies such as rheumatoid arthritis and cause considerable disability. However, little is known about the nature and extent of foot complaints in systemic lupus erythematosus (SLE) as highlighted by a recent systematic review [1]. We set out to explore the clinical features and symptoms of foot involvement among people with SLE from the patients' perspective.

## Methods

We developed a new 40-item item self-administered questionnaire, posted to patients with SLE attending adult rheumatology clinics at Auckland and Counties Manukau District Health Boards, Auckland, New Zealand. The questionnaire enquired about symptoms of foot pain, extra-articular features, assessed the anatomical distribution of symptoms according to validated foot-mannequins and considered the impact of symptoms on subjects activities of daily living and well-being.

## Results

A total of 107 responses were received, with a mean age of 52 years (SD 14.1) and a mean duration of positive diagnosis of SLE of 12 years (SD10.9). Overall 79% of respondents reported foot pain caused by SLE with 50% reporting current foot pain. All regions of the feet were affected, with the midfoot, hindfoot and ankles most troublesome. Other symptoms including swelling, numbness. soft tissue pain and cold feet were common. For 38% foot pain stopped them sleeping and in 35% foot pain negatively affected them emotionally; overall for 64% foot pain generally adversely affected their lives. Only 27% reported social and/or family activities were never impacted by foot complaints.

## Conclusions

For the first time we have characterised foot complaints in a large group of people with SLE. Foot symptoms in SLE are common, severe and heterogeneous in nature, typically having a considerable negative impact on patient's well-being.
